# Correlation of exosomal microRNA clusters with bone metastasis in non-small cell lung cancer

**DOI:** 10.1007/s10585-020-10062-y

**Published:** 2020-11-24

**Authors:** Xiao-Rong Yang, Can Pi, Ruoying Yu, Xiao-Jun Fan, Xiao-Xiao Peng, Xu-Chao Zhang, Zhi-Hong Chen, Xue Wu, Yang Shao, Yi-Long Wu, Qing Zhou

**Affiliations:** 1 Guangdong Lung Cancer Institute, Guangdong Provincial Key Laboratory of Translational Medicine in Lung Cancer, Guangdong Provincial People’s Hospital & Guangdong Academy of Medical Sciences, School of Medicine, South China University of Technology, Guangzhou, China; 2 Geneseeq Technology Inc., Toronto, ON Canada; 3 Nanjing Geneseeq Technology Inc., Nanjing, Jiangsu China; 4 School of Public Health, Nanjing Medical University, Nanjing, Jiangsu China

**Keywords:** Plasma-derived exosomal microRNAs, NSCLC, Bone metastasis, Wnt/β-catenin pathway, WGCNA

## Abstract

20–40% of lung cancer patients develop bone metastasis (BM) with significantly decreased overall survival. Currently, BM is mainly diagnosed by computerized tomography (CT) scan or magnetic resonance imaging (MRI) when symptom develops. Novel biomarkers with higher prediction value of BM are needed. Plasma-derived exosomal microRNAs had been isolated and sequenced of total 30 non-small cell lung cancer (NSCLC) patients including 16 with bone metastasis and 14 without bone metastasis. Hierarchical clustering based on the total miRNA profile can clearly separate cancer patients and healthy individuals (H), but not patients with (BM +) or without (BM−) BM. Weight Co-expression network of miRNAs (WGCNA) analyses identified three consensus clusters (A, B, C) of highly correlated miRNAs, among which cluster B (144 miRNAs) showed significantly differential expression in lung cancer patients, especially in BM + group. Pathway analysis of cluster B miRNAs revealed enrichment in metabolic pathways that may involve in preconditioning of the metastatic niche. Three differentially expressed miRNAs between BM + and BM− patients within cluster B were identified as miR-574-5p, a suppressor of Wnt/β-catenin pathway, was down-regulated, while miR-328-3p and miR-423-3p, two activators of the same pathway, were up-regulated in BM + patients. Cluster A miRNAs (n = 49) also showed trend of upregulation in BM + patients. Interestingly, pathway analysis indicated that 43 of them are associated with chromosome14, which has been suggested to promote epithelial-mesenchymal transition (EMT) and bone metastasis.

**Electronic supplementary material:**

The online version of this article (10.1007/s10585-020-10062-y) contains supplementary material, which is available to authorized users.

## Introduction

Lung cancer is the leading cause of cancer death worldwide and non-small cell lung cancer (NSCLC) which account for 80% of lung cancers [1] is one of the most common tumors metastasizing to bone. Bone is a common site of blood metastasis and the incidence of bone metastasis (BM) in NSCLC during disease course is about 30% to 40% [2]. In NSCLC, the 3-year overall survival ratio significantly decreases from 71.6 to 46.8% when tumor cells spread to bone [3].

Bone metastasis is a multi-steps process which exhibited a unique set of skeletal complications, including bone pain, pathologic fractures, hypercalcemia and spinal cord compression [4]. During the process, invasive tumor cells may intrude into the blood vessel as single circulating tumor cells (CTCs) which is facilitated by epithelial-mesenchymal transition (EMT) and the primary tumor microenvironment. In blood circulation, CTCs aggregate with platelets to survive and adhere to the bone marrow endothelium, and then extravasate into bone marrow parenchyma. Once the tumor cells in the bone marrow are reactivated from the dormancy state due to certain favorable situation, the micro-metastasis form and eventually lead to overt metastasis [5]. The advanced stages of bone metastasis can be classified into two different types: osteoblastic metastasis and osteolytic metastasis. In NSCLC patients, majority of cases is osteolytic bone metastasis [6, 7]. Osteolysis or bone breakdown is formed as a result of the disruption in the normal balance of bone resorption and formation and the exact mechanism is not fully understood [8].

Currently, Bone metastasis in lung cancer is mainly detected by skeletal scintigraphy, computerized tomography (CT), Positron emission tomography–computed tomography (PET-CT), magnetic resonance imaging (MRI). Skeletal scintigraphy (also named bone scan) which enables visualization of local bone turnover with labeled phosphonates showed best detection in marked reactive hypermetabolism of bone and relatively insensitive for tumors cause osteolysis which is most common in NSCLC [7]. CT is highly sensitive for both osteolytic and osteoplastic bone lesions, but less sensitive for tumors restricted to the marrow space [9]. Whole-body MRI is now the most sensitive and specific methods for the detection of bone-marrow metastases and extraosseous tumor extension. PET-CT is whole-body imaging modality based on metabolic or biochemical activity. Thus PET-CT can’t differentiate bone metastases from non-specific bone lesions [10]. The reported pooled sensitivity and specificity for the detection of bone metastasis by MRI were 90.6% and 95.4% on per-patient basis [9]. However, MRI equipment is expensive to purchase, maintain and operate. In most cases, patients were diagnosed using a combination of bone scan, CT and MRI. Therefore, novel methods with higher sensitivity and specificity are needed for quick and easy detection of bone metastasis.

MicroRNAs (miRNAs) which are small 18 to 24 nucleotides non-coding RNAs target the 3′ untranslated region of messenger RNAs (mRNAs) and regulate gene expression, resulting in mRNA cleavage or suppression of protein translation [11]. MiRNAs often located in the fragile regions of the chromosome which have a high frequency of deletions, rearrangements and amplification and have been implicated in malignancy [12]. At present, miRNAs are being studied as diagnostic and prognostic biomarkers for many cancers including NSCLC [13, 14], breast cancer [15], prostate cancer [16], hepatocellular cancer [17]. Many observations also strongly implicated the possibility of developing miRNAs as non-invasive circulating biomarker for the early detection of solid cancers [18, 19]. Plasma exosomes which can be released by most cell type especially cancer cells are small membrane vesicles with a diameter of 30–100 nm. Exosomes present in various body fluids (plasma, urine, saliva and other malignant effusions) and act as a mediator of cellular communication to transfer functional miRNAs, protein and mRNA to neighboring cells. The concentration of circulating exosomes is usually higher in cancer patients compared to healthy controls, increasing as the tumor progresses [20]. Moreover, exosomal miRNAs have been involved in the regulation of cell maturation, proliferation and differentiation and found to be potential biomarker for immunotherapy [21, 22]. MiRNAs in plasma exosomes are ideal for clinical detection for many reasons: miRNAs are very stable in body fluid. The structure of exosomes released by cells into the blood further protects miRNAs from degradation. Second, next generation sequencing has provided a highly robust and accuracy system for detection of miRNAs in body fluid on a genome-wide scale [23]. In this study, we focus on the miRNAs in plasma-derive exosomes to investigate the potential of using miRNAs as biomarkers for early detection of bone metastasis in NSCLC patients.

## Methods

### Patients and clinical sample collection

A total of 30 *EGFR/ALK* positive NSCLC patients were enrolled in this study including sixteen phase IV patients with bone metastasis and fourteen phase IV patients without bone metastasis (Supplementary Table 1). Among them, 25 patients (83%) were diagnosed with adenocarcinoma (ADC). The median age at diagnosis is 55, ranging from 35 to 78. There were nine males (30%), 20 (67%) females and one unknown sex type. Four patients (13%) were Stage IVa NSCLC and 26 patients (87%) were Stage IVb NSCLC. Three patients (10%) were smokers and 14 patients (47%) were never-smokers. The smoking history was unavailable in thirteen patients. Peripheral blood was collected from each patient on a regular basis from routine clinical care, and plasma sample was prepared within 2 h of blood drawn and then stored at − 80 °C. Additionally, plasma samples from 14 healthy donors were collected as control group.

### Plasma exosome isolation

1 mL of plasma sample was centrifuged at 10,000×*g* for 30 min at 4 °C to remove any cell debris. The collected supernatant was then subjected for ultra-highspeed centrifugation at 150,000×*g* for 70 min at 4 °C. Pellet containing exosome was resuspended in 200 μL PBS for downstream applications. Western blot characterization of exosome preparation was shown in Supplementary Figure S1. The presence of exosomal markers, CD63 and CD9, was confirmed by western blot.

### Exosomal RNA isolation and small RNA sequencing

Total RNA including miRNA was extracted from plasma-derived exosome using miRNeasy Serum/Plasma Kit (QIAGEN) following manufacturer’s instructions. The quantification and size distribution of the extraction were analyzed by Qubit 4.0 and Agilent Bioanalyzer 2100 (Agilent), respectively. Quantified miRNA was subjected for sequencing library preparation using NEBNext® Small RNA Library Prep Set for Illumina® (NEB Biolabs) following manufacturer’s instructions. Briefly, isolated miRNA was subjected for 3′ and 5′ adaptor ligation, followed by 17 cycles of PCR amplification. PCR products from library preparation were subjected for gel electrophoresis on 6% Novex® TBE PAGE gel (Thermo Fisher Scientific) and DNA fragments between 140 and 150 bp were recovered from the gel. Purified miRNA cDNA library was quantified by Qubit 4.0 and the size distribution was analyzed on Agilent Bioanalyzer 2100. miRNA cDNA libraries from different plasma samples were pooled and sequenced on Illumina HiSeq4000 platform.

### miRNA-seq data analysis

miRNA identification and reads counting in each miRNA were performed using miRDeep2 [24]. After trimming the 3′ adaptor sequence, all sequences ranging in length from 18 to 26 nt were recorded in a non-redundant file along with reads count. To identify known miRNAs, the miRNA tags were aligned against miRNA precursor sequences reported in the miRNA database ‘miRBase’ (release 21) using the ‘quantifier.pl’ script within miRDeep2. Differential expression (DE) analysis of miRNA sequence data was performed with the Bioconductor package edgeR [25]. miRNAs with read counts per million mapped reads (CPM) ≥ 2 in at least 20% of all samples were identified as expressed miRNAs. DE between different groups was evaluated by fitting a negative binomial generalized linear model and then adjusting the P-value for multiple testing using the Benjamini–Hochberg correction with a false discovery rate of 0.1 and a minimum log_2_(CPM) of 4.

### Weight co-expression networks

Weight co-expression network of miRNAs was performed in accordance to the protocol of WGCNA package in the R language [26]. MiRNAs were aggregated into modules by hierarchical clustering and refined by the dynamic tree cut algorithm. Thereafter, module eigenvalues were calculated. The eigenvalue is the first principal component of the miRNA expression profile within a module, representing average module expression profile. The statistical significance of module eigenvalues among the groups was accessed by Kruskal–Wallis test.

## Result

### NSCLC patients exhibited a unique miRNA profile compared to healthy population

In this retrospective study, 37 plasma samples were subjected for exosome purification and miRNA-seq. Among them, 23 samples were from 16 phase IV patients with bone metastasis (BM+) and 14 samples from 14 phase IV patients without bone metastasis (BM−). Raw reads of miRNA-seq from plasma samples were normalized to counts per million (CPM) and 1287 miRNAs were retained as expressed miRNAs in plasma exosomes (Supplementary Table 2). Using exact test, 91 miRNAs were identified as differentially expressed miRNAs (DE miRNAs) between H and C including 71 up-regulated miRNAs and 20 down-regulated miRNAs in patient group (Supplementary Table 3). Based on the DE exosomal miRNA profile, samples of healthy individuals (H) could be separated from samples of NSCLC patients (C) using supervised hierarchy clustering (Supplementary Figure S2). All these data proved that NSCLC patients exhibited a unique miRNA profile compared to healthy population.

### Detection of co-expression clusters in exosomal miRNAs

To characterize the correlation pattern and predict the function of miRNAs, we applied weighed gene co-expression network analysis (WGCNA) to the 1287 miRNAs detected in the plasma exosomes. WGCNA is widely used in genomic data analysis which can detect clusters of highly correlated genes based on pairwise correlations [26]. As shown in Supplementary Figure S3, we identified three clusters of co-expressed miRNAs which were represented by different color codes (Brown: cluster A; Turquoise: cluster B; Blue: cluster C). Cluster B had 144 miRNAs which is the largest cluster among three. 49 miRNAs were assorted into Cluster A while 95 miRNAs were in Cluster C (Supplementary Table 4).

**Fig. 1:**
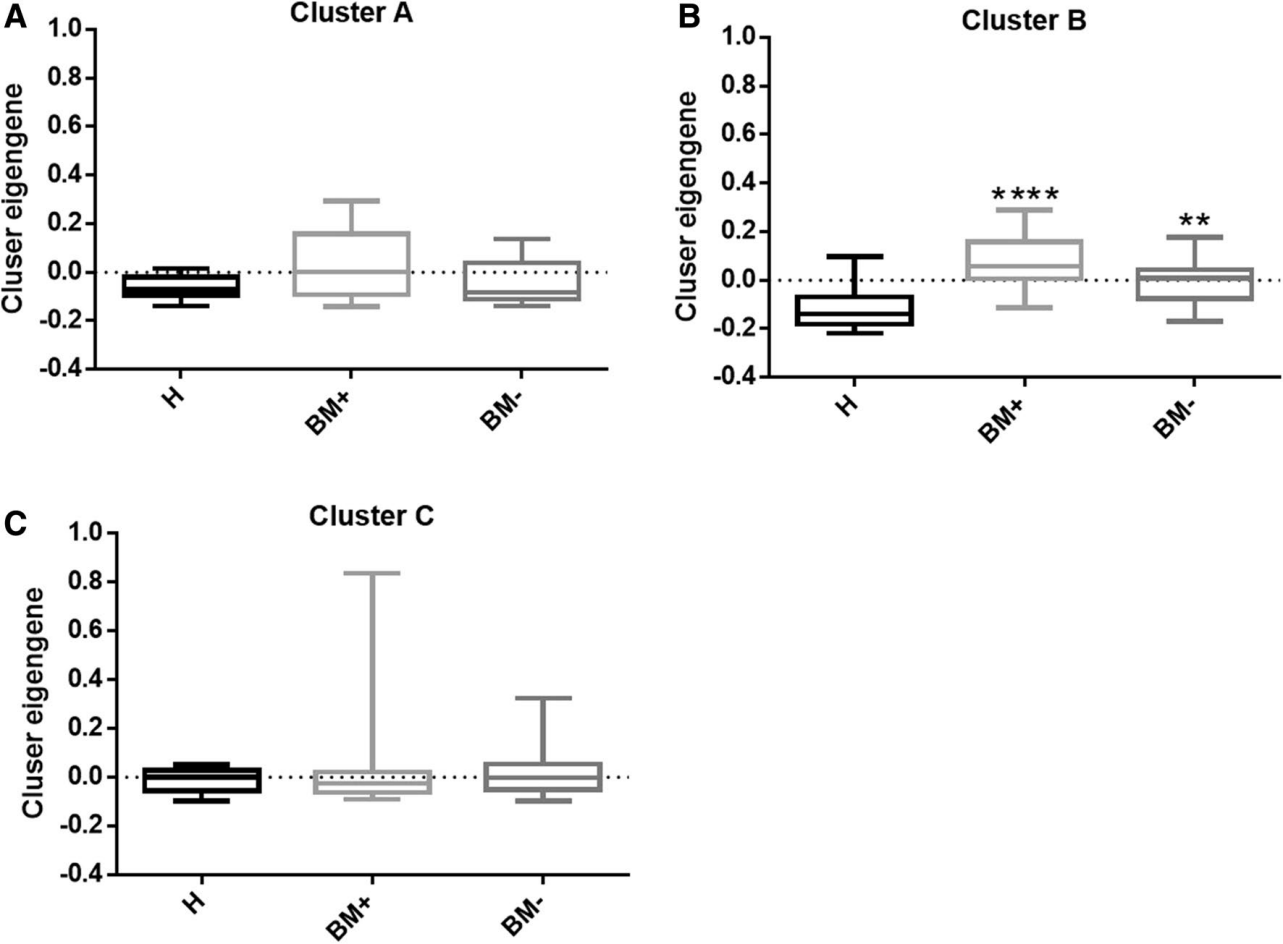
Boxplots of eigengene values across three identified modules. The eigengene values in healthy population, BM + and BM− group in three identified modules were shown in (**a**–**c**). The significance among the groups was calculated using Kruskal–Wallis test. Each column represented one group and the error bar indicated the S.E.M.**P < 0.01; ****P < 0.001.

**Fig. 2:**
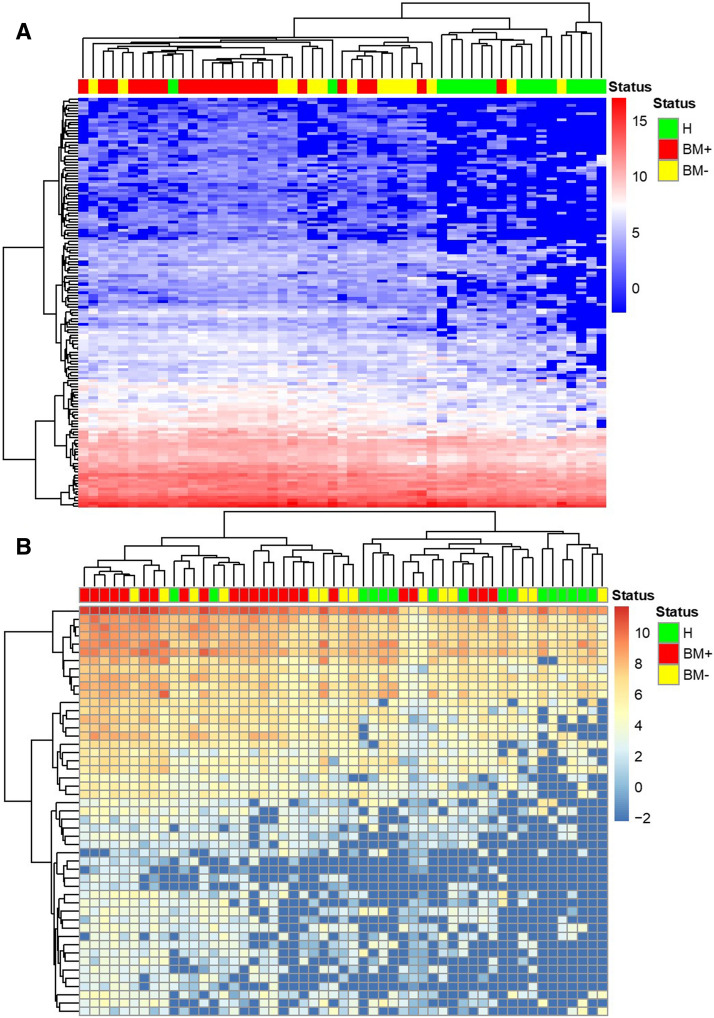
Unsupervised hierarchical clustering based on clustered miRNAs **a** unsupervised hierarchical clustering based on 144 miRNAs from cluster B. **b** Unsupervised hierarchical clustering based on 49 miRNAs from cluster A

Furthermore, BM+ group showed significant up-regulation in cluster B eigengene value compared to healthy population and BM− group which suggested miRNAs in cluster B might related to the initial of bone metastasis (Fig. 1b). The 144 miRNAs from cluster B basically differentiated BM + group from BM− group using unsupervised clustering which also shed light on the function of the miRNA cluster in bone metastasis (Fig. 2a). We performed pathway analysis of the 144 miRNAs in cluster B using miRNA enrichment analysis and annotation (MiEAA) [27] and top 20 miRNA pathways were shown in Table 1. Interestingly, cluster B was enriched in metabolism processes such as pyruvate metabolism (p = 0.010575), glycolysis and gluconeogenesis (p = 0.014983), purine metabolism (p = 0.014983), propanoate metabolism (p = 0.014983) and pyrimidine metabolism (p = 0.027086) (Supplementary Table 5).

**Table 1:** Pathway analysis of miRNAs from cluster B

Top 20 miRNA Pathways related to cluster B miRNAs	p-value	Expected	Observed
hsa00620 Pyruvate metabolism	0.010575	13.9162	28
WP534 Glycolysis and Gluconeogenesis	0.014983	18.7784	32
hsa00010 Glycolysis Gluconeogenesis	0.014983	19.6168	34
hsa00230 Purine metabolism	0.014983	20.2874	34
hsa00640 Propanoate metabolism	0.014983	10.5629	22
hsa01100 Metabolic pathways	0.014983	42.0838	57
hsa03040 Spliceosome	0.014983	25.3174	40
hsa04930 Type II diabetes mellitus	0.014983	17.6048	31
P00049 Parkinson disease	0.015597	24.479	38
WP357 Fatty Acid Biosynthesis	0.015597	11.9042	23
WP411 mRNA processing	0.015597	26.1557	40
hsa04640 Hematopoietic cell lineage	0.015597	11.2335	22
hsa04672 Intestinal immune network for IgA production	0.015597	5.7006	14
WP383 Striated Muscle Contraction	0.017108	12.2395	23
hsa04960 Aldosterone regulated sodium reabsorption	0.017108	14.5868	26
hsa05012 Parkinsons disease	0.018074	19.6168	32
hsa00061 Fatty acid biosynthesis	0.021564	5.36527	13
hsa03018 RNA degradation	0.021564	14.9222	26
hsa05010 Alzheimers disease	0.022708	26.1557	39
P02772 Pyruvate metabolism	0.024846	7.54491	16

Top20 miRNA pathways were listed in the table based on p value

In cluster A, BM + group exhibited a trend of increase in eigengene value compared to healthy population and BM− group (Fig. 1a). miRNA enrichment analysis was done with 49 miRNAs in cluster A, however, not many related pathway was found. Nevertheless, we discovered that 43 out of 49 cluster A miRNAs were expressed by chromosome 14 (Supplementary Table 6).

In cluster C, the eigengene values were comparable within healthy population group, BM+ and BM− group (Fig. 1c) which indicated that this cluster was not related to bone metastasis. The cluster A miRNAs failed to differ BM + from BM− with unsupervised clustering in Fig. 2b also suggested that cluster C miRNAs were not involve in the bone metastasis. Related pathways of cluster C were shown in Supplementary Table 7. miRNAs in cluster C seemed to related to all kinds of signaling pathways including cell cycle (p = 0.000322), proteasome and lysosome (p = 0.000322), p53 signaling pathway (p = 0.00037), insulin signaling pathway (p = 0.000322) and Ras pathway(p = 0.00037).

### Identified differentially expressed miRNAs between BM + and BM− group as potential biomarker for bone metastasis

**Fig. 3:**
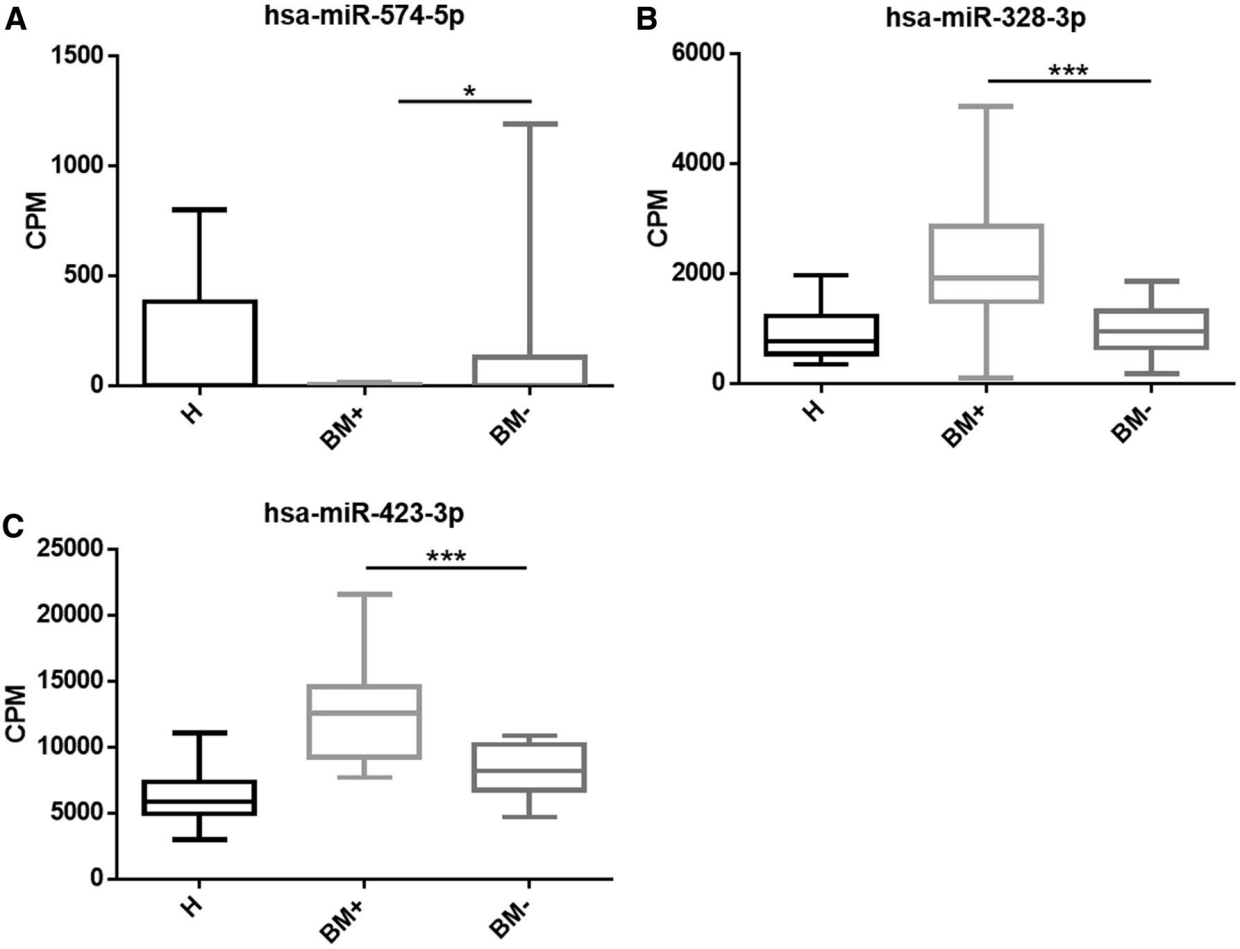
hsa-miR-574-5p, hsa-miR-328-3p and hsa-miR-423-3p were significantly up-regulated in BM+ groups. Box plot showing the individual CPM of hsa-miR-574-5p (**a**), hsa-miR-328-3p (**b**), hsa-miR-423-3p (**c**) in each sample group. P value was calculated using t-test. H: Healthy population; BM+ : samples from patients with bone-metastasis; BM−: samples from patients without bone-metastasis. Each column represented one group and the error bar indicated the S.E.M.*p < 0.05; ***p < 0.0001

Using exact test, we were able to identify differentially expressed miRNAs between BM + and BM− group. With cut off at logCPM > 4, p < 0.05, False Discovery Rate (FDR) ≤ 0.1, majority of miRNAs were excluded except hsa-miR-574-5p, hsa-miR-328-3p and hsa-miR-423-3p (Table 2). The CPM of three miRNAs in each group was shown in Fig. 3. Hsa-miR-574-5p was significantly down-regulated in BM + (Fig. 3a). Hsa-miR-328-3p and hsa-miR-423-3p were significantly up-regulated in BM + compared to BM− group (Fig. 3b, c). More importantly, the three DE miRNAs we identified, hsa-miR-574-5p, hsa-miR-328-3p and hsa-miR-423-3p, all belonged to the cluster B which is related with bone metastasis (Supplementary Table 3, Turquoise). These DE miRNAs might regulate cancer metastasis through Wnt/β-catenin signaling pathway and might be potential biomarker for bone metastasis in NSCLC.

**Table 2:** DE miRNAs identified between BM+ and BM−

DE miRNAs between BM+ and BM−	logFC	logCPM	*p*Value	FDR
hsa-miR-574-5p	5.464011	6.253159	1.91E−06	0.00246
hsa-miR-328-3p	− 1.01861	10.59402	0.000128	0.065799
hsa-miR-423-3p	− 0.65545	13.27472	0.000153	0.065799
hsa-miR-4459	3.562175	1.031715	0.000989	0.285606
hsa-miR-4763-3p	4.337457	1.317247	0.00111	0.285606
hsa-miR-877-5p	− 0.91572	7.217131	0.001579	0.323115
hsa-miR-744-5p	− 0.77977	12.15367	0.001757	0.323115
hsa-miR-4436a	3.557953	1.084548	0.002867	0.392617

Differentially expressed miRNAs between PD and DC patients were analyzed by exact test (logCPM > 4, p < 0.05, FDR ≤ 0.1)

## Discussion

In this retrospective study, we analyzed the plasma-derived exosomal miRNAs from stage IV NSCLC patients with or without bone metastasis. A total of 1287 miRNAs were identified and assorted into three major clusters A, B, C by WGCNA. Cluster A had a trend of increase in BM+ group compared to BM− and majority of cluster A miRNAs was transcripted by chromosome14. Cluster B showed significant difference between BM+ and BM− and the three DE miRNAs (hsa-miR-574-5p, hsa-miR-328-3p and hsa-miR-423-3p) identified between BM+ and BM− belonged to cluster B as well. Pathway analysis revealed that cluster B miRNAs were mostly related to metabolism processes. Hsa-miR-574-5p, hsa-miR-328-3p and hsa-miR-423-3p were reported actively involved in the Wnt/β-catenin signaling pathway and might be candidates as biomarkers for bone metastasis in NSCLC patients. Cluster C showed no difference among healthy population, BM+ and BM−, thus was not relate to bone metastasis in lung cancer.

The metabolic processes we found associated with cluster B miRNAs were highly associated with tumor metastasis. In general, metabolism changes often accompany tumor progression and were known to be associated with establishment of metastatic niches which are preconditioned for the arrival of metastatic disseminated cancer cells [28]. On the other hand, cancer cells undergo metabolic alteration and acquire metastatic traits to adapt to multiple environments. Upregulation of glycolysis [29], pyruvate kinase [30], pyrimidine phosphorylase [31] was observed in many cancers and played important role in tumor metastasis and aggressiveness. Recently, purine signaling pathway has been reported to contribute to the bone marrow metastasis in neuroblastoma [32]. All these evidences supported our finding that cluster B was metastatically relevant.

Wnt/β-catenin signaling pathway plays an important role in the epithelial-to-mesenchymal transition (EMT) and contribute to cancer progression and metastasis in different types of malignancies. MiRNAs are the major regulators of Wnt/β-catenin signaling pathway which make them ideal for therapeutic targets against metastatic tumor [33]. Here, we reported three differential expressed miRNAs which might be signatures for bone metastasis in NSCLC. Intriguingly, hsa-miRNA-574-5p, a suppressor of Wnt/β-catenin signaling pathway, has been reported to promote metastasis of NSCLC [34]. Moreover, hsa-miR-574-5p was also found to be highly related to development and metastasis of other cancer including thyroid cancer [35], colorectal cancer [36, 37] and breast cancer [38]. Both hsa-miR-328-3p and hsa-miR-423-3p were reported as activators in Wnt/β-catenin signaling pathway and promoted cancer cell invasion and metastasis in advanced non-small cell lung cancer [39] and colorectal cancer [40]. Meanwhile, hsa-miR-328 was implicated in the high glucose-induced EMT [41] and hsa-miR-423-3p was considered as potential biomarker for lung cancer diagnosis [42]. These three miRNAs might act together through Wnt/β-catenin pathway to promote EMT and could be unique makers for bone metastasis in lung cancer. The detection of these miRNA biomarkers in plasma exosomes has the potential to become a specific, sensitive and non-invasive method for monitoring bone metastasis in clinical setting for it only require blood sample which is easy to obtain. Although the miRNA profile from healthy donors and strict cutoffs have been employed to better screen for bone-metastasis-related miRNA, further studies and validation are warranted to address the role of these BM-associated miRNAs as biomarkers or therapeutic targets for advanced NSCLC patients. Meanwhile, a comparison of plasma exosomal miRNA profiles from lung cancer patients with different metastatic location would also be of great interest to validate the specificity of these miRNA biomarkers. However, we have not found any related online database thus far. Long-term monitoring of potential exosomal miRNA biomarkers as well as the study of metastatic location-related exosomal miRNA profile are warranted to further address these unanswered questions.

Overall, the 43 miRNAs from cluster A which were transcripted by chromosome 14 showed increasing expression in BM+ group. One possibility of this increase in BM+ might due to amplification of chromosome 14 in stage IV patients with bone metastasis. However, ctDNA or tumor samples from patients were not available for copy number variation detection to confirm this hypothesis. So far, chromosome 14 amplification was not reported in NSCLC or other cancers. But there was study on chromosome 14 allelic loss which is common in nasopharyngeal carcinoma and essential tumor suppressor gene loss in tumorigenesis [43].

To sum up, by comparing the plasma-derived exosomal miRNA features of NSCLC patients with or without bone metastasis, we identified a cluster of bone-metastasis related miRNAs and three DE miRNAs which might be applied to prediction of bone metastasis in NSCLC patients in future.

## Electronic supplementary material

Below is the link to the electronic supplementary material.

Online Resource 1. Figure S1: Plasma-derived exosomal miRNAs separated healthy control samples from EGFR/ALK positive NSCLC patient samples, Figure S2: Cluster dendrogram was generated by hierarchical clustering based on dissimilarity measure of genes. Supplementary file1 (DOCX 209 KB)

Online Resource 2. Table S1: clinicopathological information of patients enrolled. Table S2:Raw reads of miRNA-seq from plasma samples were normalized to counts per million (CPM) and 1287 miRNAs were retained as expressed miRNAs in plasma exosomes, Table S3: 91 differentially expressed miRNAs between healthy population and EGFR/ALK positive NSCLC patient, Table S4: The miRNAs assorted into each cluster using WGCNA, Table S5: Pathway analysis of 144 miRNAs from Cluster B, Table S6: Pathway analysis of 49 miRNAs from Cluster A, Table S7: Pathway analysis of 95 miRNAs from Cluster C. Supplementary file2 (XLSX 614 KB)

## Data Availability

All data analyzed during this study are included either in this article or in the additional files.
