# Supplementary material for: Correlation of exosomal microRNA clusters with bone metastasis in non-small cell lung cancer
Source: Clin Exp Metastasis. 2020 Nov 24;38(1):109–17. doi: 10.1007/s10585-020-10062-y (PMC7882559; doi:10.1007/s10585-020-10062-y)

Supplementary Information

**Figure S1. Biochemical characterization of exosome preparations.** 1mL of plasma sample was centrifuged at 10,000 g for 30 min at 4^o^C to remove any cell debris. The collected supernatant was then subjected for ultra-highspeed centrifugation at 150,000 g for 70 min at 4^o^C. Pellet containing exosome was resuspended in 40 μL PBS for western blot detection.

**
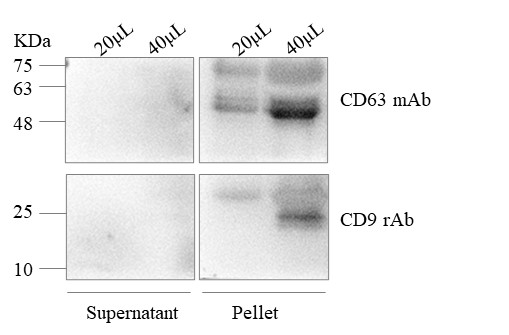
**

**Figure S2. Plasma-derived exosomal miRNAs separated healthy control samples from *EGFR*/*ALK* positive NSCLC patient samples.** Supervised hierarchical clustering of the 188 differentially expressed miRNA expression profiles from plasma exosome clearly separated healthy control samples from *EGFR/ALK* positive NSCLC patient group.


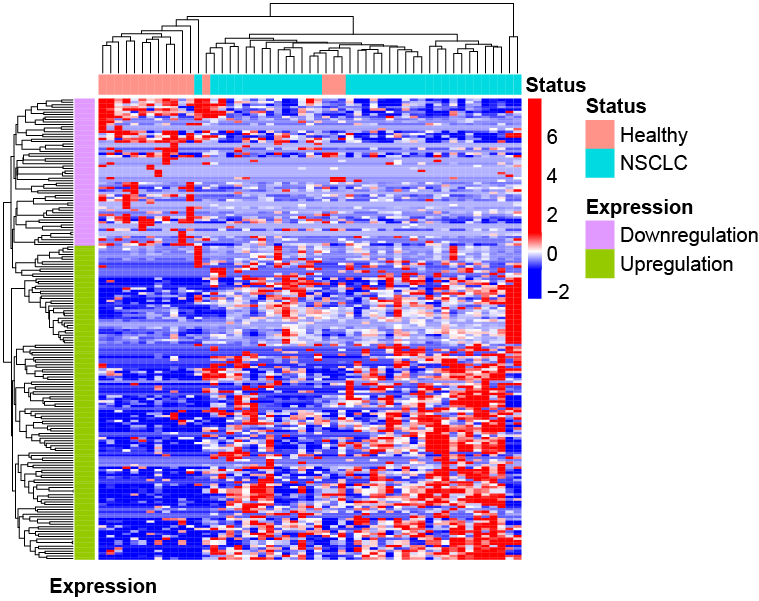


**Figure S3. Cluster dendrogram was generated by hierarchical clustering based on dissimilarity measure of genes.** The branches correspond to modules of highly interconnected groups of genes. Colored bars below the dendrogram represent the original modules. Three modules (A, B, C) were identified by the Dynamic Tree Cutting method. Each module was assigned a color as an identifier. A. Brown; B. Turquoise; C. Blue.


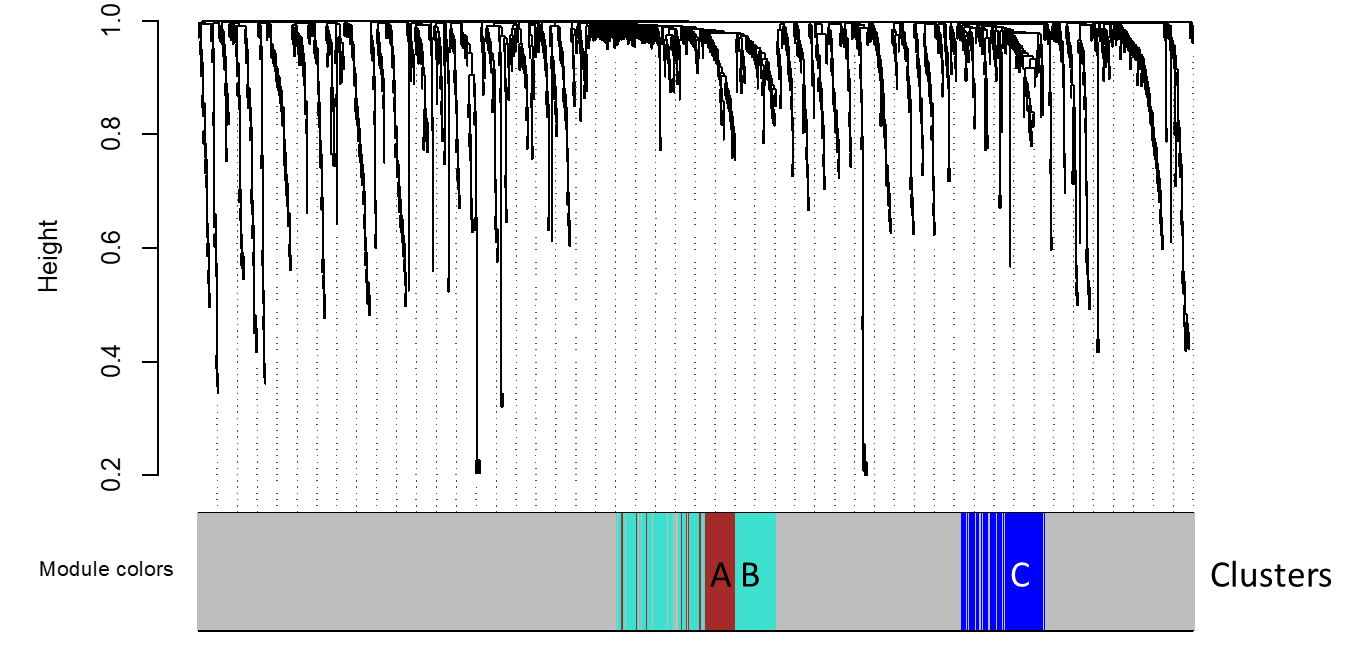

Supplement: Supplementary file 1 — Online Resource 1. Figure S1: Plasma-derived exosomal miRNAs separated healthy control samples from EGFR/ALK positive NSCLC patient samples, Figure S2: Cluster dendrogram was generated by hierarchical clustering based on dissimilarity measure of genes. Supplementary file1 (DOCX 209 KB) [file 10585_2020_10062_MOESM1_ESM.docx]
